# Predictive value of early postoperative IOP and bleb morphology in Mitomycin-C augmented trabeculectomy

**DOI:** 10.12688/f1000research.12904.2

**Published:** 2017-12-19

**Authors:** Hamed Esfandiari, Mohammad Pakravan, Nils A. Loewen, Mehdi Yaseri

**Affiliations:** 1 Ophthalmic Research Center, Shahid Beheshti University of Medical Sciences, Tehran, Iran; 2 Department of Ophthalmology, School of Medicine, University of Pittsburgh, Pittsburgh, PA, USA; 3 Ophthalmic Epidemiology Research Center, Shahid Beheshti University of Medical Sciences, Tehran, Iran; 4 Department of Epidemiology and Biostatistics, School of Public Health, Tehran University of Medical Sciences, Tehran, Iran

**Keywords:** trabeculectomy, mitomycin C, bleb morphology, Indiana bleb appearance grading scale, intraocular pressure

## Abstract

**Background**: To determine the predictive value of postoperative bleb morphological features and intraocular pressure (IOP) on the success rate of trabeculectomy.

**Methods**: In this prospective interventional case series, we analyzed for one year 80 consecutive primary open angle glaucoma patients who underwent mitomycin-augmented trabeculectomy. Bleb morphology was scored using the Indiana bleb appearance grading scale (IBAGS). Success was defined as IOP ≤15 mmHg at 12 months. We applied a multivariable regression analysis and determined the area under the receiver operating characteristic curve (AUC).

**Results**: The mean age of participants was 62±12.3 years in the success and 63.2±16.3 years in the failure group (P= 0.430) with equal gender distribution (P=0.911). IOPs on day 1, 7 and 30 were similar in both (P= 0.193, 0.639, and 0.238, respectively.) The AUC of IOP at day 1, day 7 and 30 for predicting a successful outcome was 0.355, 0.452, and 0.80, respectively. The AUC for bleb morphology parameters of bleb height, extension, and vascularization, on day 14 were 0.368, 0.408, and 0.549, respectively. Values for day 30 were 0.428, 0.563, and 0.654. IOP change from day 1 to day 30 was a good predictor of failure (AUC=0.838, 95% CI: 0.704 to 0.971) with a change of more than 3 mmHg predicting failure with a sensitivity of 82.5% (95% CI: 68 to 91%) and a specificity of 87.5% (95% CI: 53 to 98%).

**Conclusions**: IOP on day 30 had a fair to good accuracy while bleb features failed to predict success except bleb vascularity that had a poor to fair accuracy.  An IOP increase more than 3 mmHg during the first 30 days was a good predictor of failure.

## Introduction

For several reasons, the number of trabeculectomies has sharply declined in the last decades in many developed countries ^1, 2^. More surgeons are now trained in microincisional glaucoma surgeries ^3, 4^ which can be performed sooner due to a superior risk profile ^5^; laser trabeculoplasty devices have become more affordable and are used as the first line of treatment ^6, 7^; and prostaglandin analogues are now available as generic, less expensive eye medications, further reducing the number of trabeculectomies ^6^. However, in economies categorized as developing by the International Monetary Fund ^8^ and countries not in the upper quartile of the Human Development Index ^9^, trabeculectomies remain the leading surgeries for moderate and severe glaucomas ^10, 11^. Although trabeculectomies have a considerable complication rate ^12^, they remain a common primary procedure for advanced glaucoma because they lower intraocular pressure (IOP) effectively and are not as expensive as epibulbar glaucoma drainage devices ^13^, which involve a comparable surgical effort and risk profile.

Careful patient selection and postoperative management are key to making trabeculectomies successful. Recognized risk factors are, among others, younger age, a higher baseline IOP, and inflammation ^14, 15^. While meticulous preoperative planning, intraoperative use of antifibrotics, and the surgical technique impact the outcome, the postoperative period plays an equally important role ^16, 17^ including adjustment of the frequency of postoperative visits, adjustment of steroid frequency, release or lasering of scleral flap sutures, bleb massage, and bleb needling. Yet, even with the most personalized postoperative care, the individual wound healing and tissue remodeling make predicting a successful outcome difficult. Several recent studies have tried to capture the specific wound healing by correlating the early postoperative IOP or the bleb morphology with long-term outcomes, and found a low early postoperative IOP produces favorable long-term outcomes ^18– 21^. In comparison, bleb grading systems are more complex, less objective but hold promise to describe data otherwise difficult to quantify; they have not been widely adopted ^22, 23^.

The purpose of our study was to validate in our patient population the accuracy of the early postoperative IOP and bleb morphology (Indiana Bleb Appearance Grading Scale (IBAGS)) as a test to predict a failure of trabeculectomy at 12 months. In contrast to prior studies, we also examined the role of an IOP increase during the first 30 postoperative days in predicting such failure.

## Methods

### Study design

Eighty eyes of 80 consecutive patients diagnosed with primary open angle glaucoma were included in this prospective observational study. In patients who had trabeculectomies in both eyes, the eye was chosen by randomization ^24^. The study was performed at the glaucoma clinic of the Labbafinejad Medical Center of the Shahid Beheshti University of Medical Sciences, Tehran, Iran, from September 2013 to March 2015. It was approved by the ethics committee and the institutional review board at the Ophthalmic Research Center (protocol number: IR.SBMU.ORC.REC.1393.2) and followed the tenets of the Declaration of Helsinki. Informed written consent was obtained from each participant. Inclusion criteria were equal to or above 30 years of age and progressive primary open angle glaucoma (glaucomatous optic neuropathy, retinal nerve fiber layer loss and corresponding visual field defect on standard automated perimetry) that could not be controlled medically. Exclusion criteria consisted of prior ocular surgery, manipulation of the conjunctiva, a necessity for combining trabeculectomy with cataract extraction, ocular or systemic comorbidities that could affect the procedure and study outcomes including immunodeficiency, connective tissue disease, and uncontrolled diabetes. At baseline, all patients underwent a comprehensive ophthalmic examination including determination of best-corrected visual acuity (BCVA), slit lamp examination, Goldmann applanation tonometry, gonioscopy, and fundus examination.

### Surgical technique and postoperative management

All trabeculectomies were performed by two surgeons in equal numbers (HE and MP), as described in the following ^25^. After administration of intravenous sedation, a peribulbar block was placed using 2 milliliters (ml) of 2% lidocaine (Lignidic 2%, Caspian Tamin Pharmaceutical Co., Rasht, Iran). Following lid speculum insertion and cul de sacs irrigation with povidone-iodine and normal saline solution, a 7-0 silk traction suture was placed through the superior peripheral cornea. A superior peritomy was fashioned over 1-½ clock hours, and Tenon’s capsule was dissected with Westcott scissors. A 3.0 × 4.0 mm trapezoidal half-thickness scleral flap was fashioned using a crescent knife followed by lamellar dissection of the flap 1mm into the clear cornea. After creating the scleral flap, pieces of 0.02% MMC (Mitomycin-C, Kyowa, Kogyo Company, Tokyo, Japan) soaked cellulose sponges were placed under the Tenon and conjunctiva over the scleral flap for 3 minutes. After removing the sponges and vigorously irrigating the area with 50 ml of balanced salt solution (BSS), the anterior chamber was entered with a small keratome, and a block of clear cornea was removed with a 1 mm Kelly punch followed by a peripheral iridectomy using Vannas scissors. The scleral flap was then secured using two 10-0 nylon releasable sutures. The anterior chamber was formed by injection of BSS through a sideport, and the filtration was titrated by adjusting the tension of the scleral flap sutures. The conjunctiva was closed using 10-0 nylon wing sutures. Cefazolin 50 mg (Cefazolin 500, Exir Pharmaceutical Co, Tehran, Iran) and betamethasone 2 mg (Betazone, Caspian Tamin Pharmaceutical CO., Rasht, Iran) were injected subconjunctivally into the inferior fornix, and the eye was patched.

The postoperative regimen consisted of chloramphenicol 0.5% eye drops (Sina Darou Lab. Co., Tehran, Iran) 4 times a day for 1 week and betamethasone 0.1% eye drops (Sina Darou Lab. Co., Tehran, Iran) 6 times a day, which was tapered over 8 to 12 weeks depending on the inflammation. The releasable sutures were removed as needed after 72 hours depending on bleb appearance to achieve IOPs near 10 mmHg. In most cases, the releasable sutures were removed within 4 weeks after the surgery. One removed first and if the IOP was not within the target range the other one was released during the follow up one week later. Needling was done during the postoperative period for impending failure from highly vascularized and contracting blebs. Thirty minutes after injecting 0.1 ml of 0.02% Mitomycin-C into the bleb-adjacent subtenon space a 27-gauge needle was used to reform the bleb and dissect adhesions at the slit lamp.

Before the surgery patients used glaucoma medications from all four major classes, including beta-blockers, prostaglandin analogous, alpha-2 agonists, and carbonic anhydrase inhibitors. Postoperatively, patients received the same drops that had proved effective and safe before surgery if the target IOP was not achieved. The only exception was prostaglandins analogous that we tried to avoid during the first three postoperative months.

We saw our patients on a weekly basis for one month and then monthly for 12 months. At each postoperative visit, BCVA, IOP, bleb morphology, glaucoma medications, and complications were noted. A slit lamp photograph of the blebs was obtained at each visit (Haag-Streit BX 900 slit lamp, Haag-Streit Diagnostics, Köniz, Switzerland). Two glaucoma surgeons, masked to surgeon and patient, graded the bleb images on the Indiana Bleb Appearance Grading Scale (IBAGS). The IBAGS variables included the bleb height (H0-H3), extent (E0-E3), vascularity (V0-V4), and leakage (S0-S2) ^23^. A trabeculectomy was considered successful if IOP was equal or less than 15 mmHg and more than 5 mmHg with or without glaucoma medications ^18^.

### Statistical analysis

Data was described as mean ± standard deviation (SD), median, range, 95% confidence interval (95% CI) and frequency (percentage). To evaluate for difference between the two groups we used the *t*-test, Mann-Whitney, Chi-square and Fisher exact test. A change in visual acuity before and after surgery was evaluated by the Wilcoxon signed-rank test. The predictive power of bleb variables was measured with the area under the receiver operator characteristic curve (AUC). To find the relative risk (RR, Supplementary Table 1) of failure by bleb morphology and IOP at the 1-month visit, a Cox regression with defined constant time was used. Youden’s J statistic was used to capture the performance of variables (bleb morphology, IOP) as a diagnostic test. All statistical analyses were done with the SPSS software package (IBM Corp. Released 2016. IBM SPSS Statistic for Windows, Version 24.0, Armonk, NY).

## Results

A total of 80 patients were enrolled in this study. This sample size and ratio of success and failure provided an above 90% power to detect an AUC difference of 0.5 ^26^. Forty-four eyes would have provided a power of 80% to detect an IOP change of 3 mmHg while 80 eyes allowed for a 97% power. The mean age of study participants was 62.3±13.1 years, and 51.3% of the patients were male (P=0.911). The characteristics of participating patients are presented in Table 1. The procedure was considered successful in 64 patients (80%) at 12 months follow-up.

**Table 1.** Baseline characteristics of participants (mean±SD, median (range)).

	Total	Success		P
		No (n=16)	Yes (n=64)	
**age**	62.3±13.1	63.2±16.3	62±12.3	0.430 †
**male**	41 (51.3%)	8 (50.0%)	33 (51.6%)	0.911 *
**female**	39 (48.8%)	8 (50.0%)	31 (48.4%)	
**BCVA**	0.47±0.47	0.37±0.37	0.49±0.49	0.334 ‡
**IOP**	21.8±5.0	20.4±3.3	22.1±5.3	0.171 ‡
**preoperative** **medications**	1.2±1.6	1.4±1.9	1.2±1.6	0.785 ‡
**HVF MD**	16.9 ± 5.8	-16.2 ± 5	-17 ± 6	0.645 ‡
**VCD-ratio**	0.91 ± 0.93	0.84 ± 0.12	0.92 ± 1	0.666 ‡

BCVA: Best Corrected Visual Acuity. Medications: topical glaucoma medications. IOP: Intraocular Pressure. HVF MD: Humphrey visual field mean deviation. VCD: vertical cup disc ratio. † Based on t-test. ‡ Based on Mann-Whitney test. *Based on Chi-Square test.

Preoperative medications in the no success group were 1.4±1.9 and in the success group were 1.2±1.6 (p=0.785), while the postoperative medications were 1.4 ± 0.5 in the no success group and 1.0±0.0 in the success group (p=0.04). There were no significant differences between the success and failure groups in terms of age, the number of preoperative glaucoma medications, preoperative IOP, visual field mean deviation or vertical cup-disc ratios ( Table 1). The postoperative IOP was not different between these groups at any time ( Table 2) because an IOP of lower than 5 mmHg was also counted as no success in accordance to prior bleb rating studies. When such a low IOP was considered a success in the absence of hypotony maculopathy, the average IOPs was higher at month 12 (18.9 ± 2.3 [no success] and 9.8 ± 2.1 [success], p<0.001, Supplementary Table 1).

**Table 2.** Intraocular pressure at each pre- and postoperative visit. The mean and standard deviation, median and range and area under the receiver operating characteristic curve (AUC) with lower and upper confidence interval (CI) are shown for all timepoints. A higher AUC indicates a better ability to distinguish between the two groups.

Time	No success		Success		P ‡	AUC	95% CI	
	Mean±SD	Median (range)	Mean±SD	Median (range)			Lower	Upper
Baseline	21.2±3.4	20 (17 - 28)	21.9±5.2	21 (11 - 32)	0.654	0.473	0.297	0.65
Day 1	6±2.4	6.5 (3 - 9)	7.4±2.5	8 (2 - 13)	0.193	0.355	0.147	0.562
Week 1	7.1±2.1	7 (3 - 10)	7.8±2.5	8 (4 - 18)	0.639	0.452	0.239	0.664
Month 1	9.3±4.6	10 (2 - 16)	8.4±2.4	9 (3 - 19)	0.238	0.8	0.581	1
Month 3	12.6±6.8	14 (2 - 21)	9.5±2.5	10 (5 - 17)	0.143	0.85	0.635	1
Month 6	14.1±7.7	17 (4 - 23)	9.9±2	10 (5 - 15)	0.133	0.875	0.646	1
Month 12	13.7±7.3	17 (4 - 23)	10.1±1.7	10 (6 - 15)	0.143	0.875	0.646	1

‡ Based on Mann-Whitney test.

The best corrected visual acuity did not change significantly (0.47±0.47 logMAR at baseline, 0.48±0.48 postoperatively, P=0.492). Mean IOP decreased significantly from 21.8±5 mmHg at baseline to 10.6±3.8 mmHg at 12 months (p<0.001), and 25 patients needed glaucoma medications to have their IOP within the target range. There were no intraoperative complications. The most common postoperative complication was a shallow anterior chamber, followed by bleb leakage and hyphema as summarized in Table 3.

**Table 3.** List of postoperative interventions and complications.

	Total	Success		P
		No	Yes	
Bleb leakage	6 (7.5%)	3 (18.8%)	3 (4.7%)	0.091 **
Bleb resuturing	1 (1.3%)	0 (0.0%)	1 (1.6%)	>0.99 **
Hyphema	6 (7.5%)	2 (12.5%)	4 (6.3%)	0.594 **
Shallow anterior chamber	8 (10.0%)	3 (18.8%)	5 (7.8%)	0.194 **
Hypotony	1 (1.3%)	0 (0.0%)	1 (1.6%)	>0.99 **
Bleb needling	10 (12.5%)	7 (100.0%)	2 (3.1%)	<0.001 **

** Based on Fisher exact test.‡ Based on Mann-Whitney test.

The difference between success and failure groups in a leak, repeat suture, hyphema and shallow anterior chamber were insignificant (P=0.09, 0.99, 0.59, and 0.18, respectively). Bleb needling was more frequently performed in the group that failed (P<0.001). A total of ten eyes (12.5%) underwent needling, including two eyes in the success group and eight eyes in the failure group (P=0.001). One eye experienced hypotony maculopathy. Patients with a final IOP of ≤15 mmHg had a mean day 1 IOP of 7.4±2.5 mmHg compared with 6±2.4 mmHg in those with an IOP above 15 mmHg at final follow-up (P=0.193). Respective values for day 7 14 were 7.8±2.5 and 7.1±2.1 mmHg (P=0.639), and for day 30 values were 8.4±2.4 and 9.3±4.6 mmHg (P=0.238). The AUC for IOP at day 1, day 7 and 30 as a predictor of success was 0.355, 0.452, and 0.80 respectively. The bleb morphology parameters according to the IBAGS are detailed in Table 4.

**Table 4.** Bleb morphologic characteristics at each postoperative visit.

Parameter	Time	No success		Success		P ‡	AUC	95% CI	
		Mean±SD	Median (range)	Mean±SD	Median (range)			Lower	Upper
Bleb H	Week 2	2.45±0.69	3 (1 to 3)	2.17±0.54	2 (1 to 3)	0.098	0.368	0.172	0.564
	Month 1	2.27±0.65	2 (1 to 3)	2.12±0.53	2 (1 to 3)	0.35	0.428	0.233	0.624
	Month 3	1.64±0.67	2 (1 to 3)	1.96±0.4	2 (1 to 3)	0.024	0.652	0.444	0.859
	Month 6	1.36±0.5	1 (1 to 2)	1.78±0.54	2 (1 to 3)	0.017	0.691	0.523	0.859
	Month 12	1.36±0.5	1 (1 to 2)	1.74±0.56	2 (1 to 3)	0.038	0.669	0.5	0.838
Bleb E	Week 2	2.55±0.52	3 (2 to 3)	2.36±0.48	2 (2 to 3)	0.25	0.408	0.224	0.592
	Month 1	2.18±0.4	2 (2 to 3)	2.3±0.49	2 (1 to 3)	0.41	0.563	0.391	0.734
	Month 3	1.73±0.65	2 (1 to 3)	2.12±0.4	2 (1 to 3)	0.012	0.669	0.47	0.869
	Month 6	1.55±0.52	2 (1 to 2)	2.09±0.37	2 (1 to 3)	<0.001	0.744	0.568	0.92
	Month 12	1.82±0.75	2 (0 to 2)	1.95±0.53	1 (0 to 1)	0.002	0.749	0.57	0.928
Bleb V	Week 2	2.18±0.6	2 (1 to 3)	2.3±0.49	2 (1 to 3)	0.53	0.549	0.357	0.74
	Month 1	1.82±0.6	2 (1 to 3)	2.19±0.52	2 (1 to 3)	0.045	0.654	0.474	0.834
	Month 3	1.64±0.67	2 (0 to 2)	1.86±0.49	2 (1 to 3)	0.368	0.565	0.38	0.751
	Month 6	1.55±0.69	2 (0 to 2)	1.54±0.53	2 (0 to 2)	0.76	0.475	0.282	0.668
	Month 12	1.36±0.67	1 (0 to 2)	1.46±0.53	1 (0 to 2)	0.702	0.532	0.338	0.725

H: height. E: extension. V: vascularization. ‡ Based on Mann-Whitney test.

The AUC for bleb morphology parameters of bleb height, extension, and vascularization on day 14 were 0.368, 0.408 and 0.549, respectively. The AUC values for day 30 were 0.428, 0.563, and 0.654. Based on the AUCs, there was no single bleb variable at month 1 to predict success at month 12.

Surprisingly, the AUC of the IOP change from day 1 to day 30 was a good predictor of success (AUC=0.838, 95% CI: 0.704 to 0.971, Supplementary Table 1). Based on Youden’s J index, the change in IOP less than or equal to 3 mmHg could predict the success in month 12 with the sensitivity of 82.5% (95% CI: 68 to 91%) and specificity of 87.5% (95% CI: 53 to 98%).

Raw data collected from all study participants Click here for additional data file. Copyright: © 2017 Esfandiari H et al. 2017 Data associated with the article are available under the terms of the Creative Commons Zero "No rights reserved" data waiver (CC0 1.0 Public domain dedication).

## Discussion

Trabeculectomy remains a common surgery to lower IOP in glaucoma patients and has a long history. In 1958, Grant was the first one to describe a guarded filtering procedure in enucleated eyes with subsequent facility measurements which he termed trabeculectomy ^27^. Sugar followed by performing the first such trabeculectomy in patients in 1961 ^28^ and Cairns finally popularized the term and technique by providing detailed drawings and figures ^29^. The procedure was eventually made more effective by adding Mitomycin-C as an antifibrotic ^30^ and somewhat titratable by using sutures that can be lasered or manually released ^31, 32^. The variable success rate of this procedure can be improved by careful patient selection, surgical technique ^33^, and postoperative management ^15^. Identifying patients at risk is critical because a timely intervention can improve the outcomes of trabeculectomy ^16^; preoperative factors including high baseline IOP, number of glaucoma medications, young age, aphakia or pseudophakia and prior surgery with conjunctival scarring are known risk factors for failure ^33^. The success and failure groups in our study have similar baseline IOP, medications, visual field loss and optic nerve damage indicating that any risk factors related to those were relatively equal in both.

Several recent studies found a low early postoperative IOP produces favorable long-term outcomes ^18– 21, 34^ but also causes more frequent and more severe complications that include a shallow anterior chamber, choroidal effusion, hypotony maculopathy, and vision loss ^35^. Although we did observe that the IOP change during the first month was a good predictor of failure, we found only a weak relationship between absolute IOP at day 1, 7, and 30 and long term success. This is similar to Polikoff *et al*. ^36^ who also reported that early post-trabeculectomy IOP is a poor predictor of success at one year follow-up. The accuracy of a test depends on how well it separates groups under study into those with and without the issue in question. Accuracy is measured by the area under the ROC curve and is commonly classified into .90-1 = excellent, .80-.90 = good, .70-.80 = fair, .60-.70 = poor and .50-.60 = fail ^26^. In our study, the AUC for IOP was the highest on day 30 with 0.8, making it a fair to good predictor ^26^. Early post-trabeculectomy IOP is affected by various factors that include reduced aqueous production, inflammation, the amount of TGF-beta and other growth factors ^37^, and breakdown of the blood-aqueous barrier, over filtration, and choroidal detachment. In our study, the IOP appears to summarize these and other factors reasonably well to a single variable that can be used as a predictor.

An advantage of this study is that approximately 75% more patients participated than in prior bleb grading studies ^22, 23, 38, 39^. Different from those, we also examined the change in IOP during the first 30 days and found that it served as a good predictor of success that performs better than the IOP itself. The cutoff for an at-risk IOP elevation was only 3 mmHg. Our study was not designed to discover causality, however. It is likely that this finding identifies patients at risk of failure who experienced an IOP increase despite an intensified treatment. It could also indicate that these were patients who received a suboptimal postoperative management. An elevated IOP will increase the stretch of the bleb wall and incite a reactive fibrosis. Stretch is a well-established activator of fibroblast proliferation and fibrosis ^40, 41^. This may lead to a cycle of auto-enhancing IOP increase with further increase of stretch and fibrosis that again elevates pressure as the bleb wall becomes thicker and the bleb contracts. In this model, IOP drives the morphological changes of the bleb. This order matches our AUC findings better (the AUC was better for IOP change than for bleb morphology) than a primary morphological change that is followed by increased IOP. Our observation is supportive of the practice to inject or needle with postoperative 5-fluorouracil or Mitomycin-C to reduce the fibrosis ^42, 43^.

Like IOP, a bleb’s morphology is a reflection of a patient’s specific wound healing that is directly observable but more difficult to quantify. The development of bleb grading scales was an attempt to approach this problem systematically ^22, 23^. While grading systems can be useful teaching tools of bleb features, its inherently subjective nature and categorical data weaken the predictive power. Accordingly, we found only low AUC values for bleb morphology that ranged from 0.368 to 0.549 on day 14 and would be interpreted as a test failure. The day 30 features would be interpreted only slightly better, as poor. Of the variables bleb height, extension and vascularization, vascularization had the highest accuracy. These findings match Picht *et al*. ^44^ who concluded an early vascularization indicates a poor prognosis with higher IOP at 12 months and may support use of anti-VEGF agents ^45, 46^. The above reasons and recent reports of ab interno trabeculectomy results with a reduced complication rate, considerable range of glaucoma indications, and results that can rival traditional glaucoma surgery ^47– 55^ suggest a conventional trabeculectomy with Mitomycin-C may be more appropriately used as a secondary procedure when microincisional surgical modalities are available or only be used in advanced glaucoma with a rather low IOP target.

In conclusion, we found that the IOP change during the first month, rather than the IOP at each visit, was a good predictor of failure. The bleb morphological features did not predict failure except for bleb vascularity which performed poorly to fairly. These finding highlight how important it is to carefully observe for IOP and bleb vascularity changes and to intervene swiftly if necessary.

## Data availability

The data referenced by this article are under copyright with the following copyright statement: Copyright: © 2017 Esfandiari H et al.

Data associated with the article are available under the terms of the Creative Commons Zero "No rights reserved" data waiver (CC0 1.0 Public domain dedication). 

**Dataset 1: Raw data collected from all study participants.** DOI, 10.5256/f1000research.12904.d181288 ^56^.

## Supplementary material

**Supplementary Table 1: Intraocular pressure at each pre- and postoperative visit when success included IOPs below 5 mmHg without complications**.
