# Supplementary material for: Predictive value of early postoperative IOP and bleb morphology in Mitomycin-C augmented trabeculectomy
Source: F1000Res. 2017 Dec 19;6:1898. Originally published 2017 Oct 27. [Version 2] doi: 10.12688/f1000research.12904.2 (PMC5747341; doi:10.12688/f1000research.12904.2)
Supplement: Supplementary file 2 [file f1000research-6-14499-s0001.tgz › 05b2f97b-33f9-4d37-938b-5b22a3399237.pdf]

Supplementary Table 1: Intraocular pressure at each pre- and postoperative visit when success included IOPs below 5 mmHg without complications.

Time	No success		Success		P‡	AUC	95% CI	
	Mean±SD	Median (range)	Mean±SD	Median (range)			Lower	Upper
Baseline	21.0 ± 3.8	20 (17 - 28)	21.9 ± 5.1	21 (11 - 32)	0.603	0.483	0.296	0.670
Day 1	6.4 ± 2.2	7 (3 - 9)	7.3 ± 2.5	8 (2 - 13)	0.442	0.409	0.194	0.625
Week 2	7.0 ± 2.2	7 (3 - 10)	7.8 ± 2.5	8 (4 - 18)	0.552	0.434	0.200	0.667
Month 1	12.3 ± 2.1	12 (10 - 16)	8.2 ± 2.6	9 (2 - 19)	0.000	0.913	0.824	1.000
Month 3	17.1 ± 3.2	16 (13 - 21)	9.3 ± 2.7	9 (2 - 17)	0.000	0.965	0.915	1.000
Month 6	19.4 ± 2.5	18 (17 - 23)	9.7 ± 2.3	10 (4 - 15)	0.000	1.000	1.000	1.000
Month 12	18.9 ± 2.3	18 (17 - 23)	9.8 ± 2.1	10 (4 - 15)	0.000	1.000	1.000	1.000

‡ Based on Mann-Whitney test.
