# Supplementary material for: Predictive value of early postoperative IOP and bleb morphology in Mitomycin-C augmented trabeculectomy
Source: F1000Res. 2017 Dec 19;6:1898. Originally published 2017 Oct 27. [Version 2] doi: 10.12688/f1000research.12904.2 (PMC5747341; doi:10.12688/f1000research.12904.2)
Supplement: Peer review history

# Peer review: Referee response for version 2

**DOI:** 10.5256/f1000research.14499.r29149

**Published:** 2017-12-27

**Authors:** Frances Meier-Gibbons

**Affiliations:** 1 Eye Center Rapperswil, Rapperswil, Switzerland

Thanks for the changes and addition, I approve the manuscript in the revised version.

I have read this submission. I believe that I have an appropriate level of expertise to confirm that it is of an acceptable scientific standard.

# Peer review: Referee response for version 1

**DOI:** 10.5256/f1000research.13988.r27865

**Published:** 2017-11-28

**Authors:** Frances Meier-Gibbons

**Affiliations:** 1 Eye Center Rapperswil, Rapperswil, Switzerland

The study is carefully done and the topic is interesting for clinicians.

 Comments/remarks:

1.  You write the exact number of preoperative and postoperative medications the patients used. As there are differences in the IOP-lowering effects of the different types of these drugs, it would be interesting to see what type of IOP-lowering drugs the patients applied and if there was a difference in the type of drugs used pre- and postoperatively. 
2. You state that you used postoperative needling with Mitomycine C, but you do not clarify when exactly ("impending failure from a contracting bleb" is not specific). Please give a comment in which patients you used the needling and add also in how many patients a needling was necessary.
3. In the "Conclusions" of the abstract you state that "the bleb features failed to predict a successful outcome" while in the "Conclusions" at the end of the manuscript you say that an augmented vascularity in the bleb should be carefully observed. Please add this sentence in the "Conclusions" of the abstract.

I have read this submission. I believe that I have an appropriate level of expertise to confirm that it is of an acceptable scientific standard, however I have significant reservations, as outlined above.

# Response

**Published:** 2017-12-01

**Authors:** Nils Loewen

**Affiliations:** University of Pittsburgh Medical Center, USA

The study is carefully done and the topic is interesting for clinicians.

Comments/remarks:

**1. **You write the exact number of preoperative and postoperative medications the patients used. As there are differences in the IOP-lowering effects of the different types of these drugs, it would be interesting to see what type of IOP-lowering drugs the patients applied and if there was a difference in the type of drugs used pre- and postoperatively. 

**Authors: **We very much appreciate the reviewer’s time and care reviewing our manuscript. Thank you for allowing us to clarify this point. We now state in Methods: "Before the surgery patients used glaucoma medications from all four major classes, including beta-blockers, prostaglandin analogous, alpha-2 agonists, and carbonic anhydrase inhibitors. Postoperatively, patients received the same drops that had proved effective and safe before surgery if the target IOP was not achieved. The only exception was prostaglandins analogous that we tried to avoid during the first three postoperative months."

**2. **You state that you used postoperative needling with Mitomycin C, but you do not clarify when exactly ("impending failure from a contracting bleb" is not specific). Please give a comment in which patients you used the needling and add also in how many patients a needling was necessary.

**Authors:** Thank you for allowing us to clarify this point. We needled only highly vascularized and contracting blebs when the IOP was uncontrolled. We now state: "A total of ten eyes (12.5%) underwent needling, including two eyes in the success group and eight eyes in the failure group (P=0.001)."

**3. **In the "Conclusions" of the abstract you state that "the bleb features failed to predict a successful outcome" while in the "Conclusions" at the end of the manuscript you say that an augmented vascularity in the bleb should be carefully observed. Please add this sentence in the "Conclusions" of the abstract.

**Authors** **: **This is a good point. We have modified the Conclusions in the Abstract based on the reviewer’s comments:

“Conclusions: The postoperative IOP on day 30 had a fair to good accuracy while the bleb features failed to predict a successful outcome except for bleb vascularity that predicted success with poor to fair accuracy.  An IOP increase by as little as more than 3 mmHg during the first 30 days was a good predictor of failure. It is important to carefully observe for IOP changes and bleb vascularity to intervene if necessary.”

# Peer review: Referee response for version 1

**DOI:** 10.5256/f1000research.13988.r27402

**Published:** 2017-11-08

**Authors:** Lin Ding

**Affiliations:** 1 Aier School of Ophthalmology, Changsha Aier Eye Hospital, Changsha, China

This is a carefully done study containing interesting results which merit indexing. It is very useful and can provide a proof for ophthalmologist to decide how long the patient should be re-visit postoperation according to IOP change. For the benefit of the reader, however, a number of points need clarifying and certain statements require further justification.  A few minor revisions are list below.

1. There are mistakes in the study results and we listed some examples in the following and you should revised it in your paper carefully and discreetly.
  1.1 In the article, they describe that collect IOP outcomes on Day 7, but it was Week 2 in table 2. In the results part of Line 3 Page 1 (All P≥0.2), but it was 0.193 in day 1 in table 2.
  1.2  IOP change more than 3mmHg in Page 1, but it was less than or equal than 3 mmHg in Page 6.
2. There are some misunderstanding in the article.
  2.1 The first sentence in the results of page 1. (Age and gender were equally distributed (62.3±13.1 years, P=0.911).)
  2.2 The anterior chamber was formed by injection of BSS a paracentesis, and the filtration was titrated by adjusting the tension of the scleral flap sutures.(Page 3).
3. The releasable sutures were removed as needed after 72 hours depending on bleb appearance to achieve IOPs near 10 mmHg. (Page 3, Marked in yellow and underline). In what time the releasable suture were removed for most of them (for example 7-10 days or something else for 70%(or ?%) patient), and you removed the two releasable suture for one time or two time respectively. If you removed the suture for two times, how much time will pass between them.

I have read this submission. I believe that I have an appropriate level of expertise to confirm that it is of an acceptable scientific standard.

# Response

**Published:** 2017-12-01

**Authors:** Nils Loewen

**Affiliations:** University of Pittsburgh Medical Center, USA

**1. **There are mistakes in the study results and we listed some examples in the following and you should revised it in your paper carefully and discreetly.

**1.1** In the article, they describe that collect IOP outcomes on Day 7, but it was Week 2 in table 2. In the results part of Line 3 Page 1 (All P≥0.2), but it was 0.193 in day 1 in table 2.

**Authors: **We very much appreciate taking the time and effort to assess our manuscript. We are grateful for the many good suggestions.

 There was indeed a discrepancy in the Results section. It should be week 1 in the table as we now write correctly. We have confirmed this in our source SPSS database file.

 Regarding the P values in the abstract, we replaced >0.2 in the sentence: 

 “IOP of patients with a successful outcome did not differ from unsuccessful ones on day 1, 7 and 30 (all P≥0.2).“ with specific P values for each time point: “IOP of patients with a successful outcome did not differ from unsuccessful ones on day 1, 7 and 30 (P= 0.193, 0.639, and 0.238, respectively.)”

**1.2 ** IOP change more than 3mmHg in Page 1, but it was less than or equal than 3 mmHg in Page 6.

**Authors: **That is correct but there is no real contradiction. The reason for this is that we talk about that a change in IOP less than or equal to 3 can predict the *success* at month 12. In contrast, we talk about *failure* when a change of more than 3 mmHg was observed.

**2. **There are some misunderstanding in the article.

**2.1** The first sentence in the results of page 1. (Age and gender were equally distributed (62.3±13.1 years, P=0.911).)

**Authors:** Thank you for pointing this out. We changed the sentence "Age and gender were equally distributed" to "The mean age of the study participants was 62±12.3 years in the success and 63.2±16.3 years in the failure group (P= 0.430) with equal gender distribution (P=0.911)".

**2.2 **The anterior chamber was formed by injection of BSS a paracentesis, and the filtration was titrated by adjusting the tension of the scleral flap sutures.

**Authors:** We apologize and have changed the sentence to state “The anterior chamber was formed by injection of BSS a paracentesis, and the filtration was titrated by adjusting the tension of the scleral flap sutures “to “The anterior chamber was formed by injection of BSS through a sideport, and the filtration was titrated by adjusting the tension of the scleral flap sutures".   

**3. **The releasable sutures were removed as needed after 72 hours depending on bleb appearance to achieve IOPs near 10 mmHg. (Page 3, Marked in yellow and underline). In what time the releasable suture were removed for most of them (for example 7-10 days or something else for 70%(or ?%) patient), and you removed the two releasable suture for one time or two time respectively. If you removed the suture for two times, how much time will pass between them.

**Authors:** We changed Methods to state: "In most cases, the releasable sutures were removed within 4 weeks after the surgery. One removed first and if the IOP was not within the target range the other one was released during at the follow up one week later. Needling was done during the postoperative period for impending failure from highly vascularized and contracting blebs".
